# Notes on Preparations for the Sick

**Published:** 1888-06

**Authors:** 


### Notes on Preparations for the Sick.

---

**Aerated Distilled Water. Sweet Coated Pills.**—B. Keen, Bristol.

The **Distilled Water** is bright and sparkling, put up in champagne bottles, and sold at a nominal price. The popularity of the various spas, British and foreign, may be considered to indicate the importance of water as a medicinal agent. When we contemplate the difficulties in the way of excretion where the kidneys are in a pathological state, when we observe the various discomforts dependent on deficient hepatic activity, and when we meet with cases where a glass of Burgundy invariably gives rise to pains, or when a glass of bitter ale is poison and stout a still more potent one, we begin to realise the possibility of a therapeutic value in distilled water. The necessity for a thorough flushing of the tubes and channels of the tissues does not at first sight appear to be urgent; but when we think of the condition of the blood and tissues at such time as when a gouty attack is imminent, it is clear that the excretory function does need the assistance of a solvent in profusion which will wash away the tissue *débris* accumulating and being deposited in the interstices. What better solvent is possible than distilled water? and, it being contrary to the instincts of civilised human nature to take any fiuid in profusion which is not retailed at a given price from a bottle, this prepared distilled water will comply with the required conditions and become acceptable. The suggestive champagne bottle may give the contents a borrowed charm which may add to the therapeutic activity.

The **Pills** have the newly discovered sweetening agent, saccharine, used in small quantity in the coating, giving them a sweetish taste. The coating is easily soluble: all the Pharmacopœia pills are kept in readiness, and eighty-seven other formulæ comprise the list of those kept in stock: any of these can be dispensed without delay. They appear to be a distinct advance on all other kinds of coated pills, and are not more expensive than those of other pill manufacturers.

---

**Essence of Beef. Concentrated Beef Tea. Meat Lozenges.**—GEO. MASON & CO., LIMITED, London.

The **Essence of Beef** appears to be of good quality; it is prepared from British meat, and consists solely of the juices of the meat, entirely free from water or any other added matter. It is entirely free from fatty matter, is clear, palatable, and nutritious: no water should be added, but it should be taken in teaspoonful doses as a jelly. The price of this Essence compares very favourably with that of the numerous other varieties of beef essence of other makers.

The **Concentrated Beef Tea** in skins forms a ready method of making good beef tea in an emergency: it is merely necessary to cut off a portion and dissolve it in boiling water, which may be done in one minute.

The **Meat Lozenges** are a portable nourishment in a most convenient form for invalids and travellers.

---

**Lloyd's Universal Food. King's Cooked Oatmeal. King's Wheaten Food.**—GEORGE KING & CO., London.

As a food for invalids, weakly children, and delicate persons, the **Universal Food** is said to be unsurpassed. It is described as consisting "of the most nutritious and finest selected cereal grains and pulse, combined with the active constituents of pure fresh malt meal, in such proportions as to render the mixture as nearly as possible chemically identical with the constitunets of the human body itself." It is said to be prepared from the formula of Dr. Domett Stone; but the prescription is withheld, and therefore we are not informed of the details of its composition. The process of cooking adopted in its manufacture is far from efficient, inasmuch as the starch-granules are for the most part unbroken and are commonly grouped together within the original unbroken cell-walls of the vegetable tissue: it must therefore be submitted to further heat before it can be suitable food for those who are unable to digest raw starch.

The **Cooked Oatmeal** is said to have the great advantage that "it requires only one moment's boiling, and having been previously perfectly cooked it is more digestible than ordinary oatmeal prepared in the usual somewhat tedious manner." Everyone knows the value of oatmeal, which is now as familiar on the breakfast table of the English as it formerly was on that of the Scotch and Irish only: the time necessary for its preparation has been the great objection to its daily use.

We have examined this preparation microscopically, and find that the starch-granules are free, but have not been broken up: they do not show any evidence of the action of heat. The powder must therefore be boiled before it can become an easily-digested and nutritious food. We do not doubt that this preparation is more quickly cooked and more easily assimilated than the ordinary oatmeal; but on the other hand, we question if the aperient action of the ordinary crushed oatmeal is retained by the more digestible, less irritating, and therefore more nutritious preparation.

The **Wheaten Food** is prepared solely from the finest wheat, and contains the most nutritive portions of the grain: it is free from any chemical addition, and "is thoroughly cooked by an improved patent process, which renders it more digestive than when cooked by the ordinary methods." We know not what this process of cooking may be, but the powder under the microscope shows the entire starch-grains uncooked, uncracked, and uninfluenced by heat: it must therefore be well boiled before it can become suitable food for infants, otherwise the starch grains will pass through the alimentary canal undigested and have little nutrient value.

---

**Dr. Michaelis' Tonic Cocoa.**—Kuhner, Hendschel & Co., London.

This is prepared by Messrs. Stollwerck Brothers, of Cologne, from pure cocoa and soluble extract of acorns. It is said to be a most efficacious remedy for catarrhal affections of the digestive organs, and is increasingly used as a substitute for coffee, tea, or chocolate.

**Griffin's Nutritive Suppositories.**—A. W. Griffin, Bath.

Since the introduction of peptonising agents into daily practice, the methods of feeding by rectum have been greatly improved; and there is this advantage with regard to rectal feeding, that a bitter taste in the product is immaterial. Many patients strongly resist our efforts to improve their nutrition by the use of peptonised foods in the natural way; but we hold that all food given by the rectum should be rendered ready for absorption by the use of peptonising agents. We have the choice between fluid enemata and solid suppositories; sometimes the one will be the better, but sometimes the other. As a rule, we believe that the fluid form is the best; and peptonised milk, with various additions according to necessity, leaves little to be desired. When, however, the rectum is irritable and refuses to retain fluids, or when the patient has an objection to the administration of the ordinary fluid enema, the solid suppository becomes indispensable and invaluable. These prepared nutritive suppositories do away with all the difficulties in the preparation of peptonised enemata: they melt at the temperature of the body, and the melted product is easily, completely and slowly absorbed. Each suppository weighs two drachms, and is equal in nutritive value to four ounces of good beef tea. They are readily introduced, do not irritate the rectum, and are retained when fluid injections come away. The difficulties associated with persistent rectal feeding are much diminished by the use of such solid concentrated nutritive suppositories as those under notice.

---

**Liq. Podophyllin (Hockin).**—Hockin, Wilson & Co., London.

The profession is well alive to the superior value of fluid preparations of this drug. The solution of the resin in spirit with tincture of ginger, first suggested by Dr. Dobell, more recently the tincture of the Pharmacopœia, have convinced the observant that the drug in fluid form is less likely to give rise to griping pains and the other violent effects which now and then follow the administration of ordinary doses in pills. The cholagogue action is more easily obtained with a less violent aperient result and with more uniformity of action with the fluid tincture, than is the case with the resin given in pills. The liquor prepared by Hockin, Wilson & Co. has the advantage of being easily miscible with water, acid or alkaline solutions, ethers, infusions, &c.; there is also the advantage that it manifests little or no aperient action. We have given this preparation in drachm doses three times daily, also in two-drachm doses each night, with no aperient result: whether the cholagogue action is in any degree impaired by the abstraction of the aperient elements of the podophyllin is not easy to prove: patients have improved whilst taking it, but they have generally also taken a saline aperient in the morning. One drachm is stated to be equal to a quarter grain of the resin: we doubt if the therapeutic effect of the two substances can be spoken of as equal, inasmuch as the resin is sometimes too active an aperient; but the liquid in corresponding dose has no such action.

---

**Pulv. Pepsinæ Comp.**—T. Buxton, Bristol.

Each twenty-grain dose of this power contains three grains of pepsine in concentrated form, with pancreatine and diastase, in combination with hydrochloric and lactic acids.

Such a tridigestive powder as this should be of use in various indefinite conditions of abnormal digestion. It must often be the case that, if one fluid is deficient in digestive power, the others will also be defective; for instance, in cases of anæmic dyspepsia, where the impoverished condition of the blood is likely to affect the quality of all the digestive juices. The powder is designed to aid such conditions as this, and we have not been disappointed with the results obtained by its use.

